# Plasmid Sequences of Four Large Plasmids Carrying Antimicrobial Resistance Genes in Escherichia coli Strains Isolated from Beef Cattle in Japan

**DOI:** 10.1128/MRA.00219-20

**Published:** 2020-05-14

**Authors:** Shiori Yamamoto, Wataru Kitagawa, Motoki Nakano, Hiroshi Asakura, Eriko Iwabuchi, Teruo Sone, Kozo Asano

**Affiliations:** a Applied Microbiology, Graduate School of Agriculture, Hokkaido University, Sapporo, Hokkaido, Japan; b Division of Biomedical Food Research, National Institute of Health Sciences, Kawasaki, Kanagawa, Japan; c Bioproduction Research Institute, National Institute of Advanced Industrial Science and Technology, Sapporo, Hokkaido, Japan; d Department of Nutrition, School of Nursing and Nutrition, Tenshi College, Sapporo, Hokkaido, Japan; Indiana University, Bloomington

## Abstract

Escherichia coli is a common reservoir for antimicrobial resistance genes that can be easily transformed to possess multidrug resistance through plasmid transfer. To understand multidrug resistance plasmids, we report the plasmid sequences of four large plasmids carrying a number of genes related to antimicrobial resistance that were found in E. coli strains isolated from beef cattle.

## ANNOUNCEMENT

Antimicrobial resistance is a serious concern in human medicine and public health (1). The commensal Escherichia coli is a common bacterial reservoir for antimicrobial resistance genes because of its ability to donate and to accept transmissible genetic elements such as plasmids. E. coli is distributed in a variety of warm-blooded animals (2, 3). Plasmids allow the rapid transfer of antimicrobial resistance genes among bacteria (4) and are thought to contribute to the recent emergence of multidrug-resistant bacteria (5, 6). We recently obtained multidrug-resistant E. coli strains from beef cattle feces in Japan (7), and large plasmids carrying antimicrobial resistance genes were considered to facilitate drug resistance in those strains (8). To understand the functionality of the large plasmids, we sequenced four plasmids that conferred multidrug resistance to E. coli strains.

Multidrug-resistant E. coli strains were grown on lysogeny broth agar, and plasmid DNA was extracted from the bacterial cultures using a NucleoBond Xtra midikit (Macherey-Nagel). Plasmid DNA was transformed into the host E. coli strain DH10B (Thermo Fisher Scientific) and purified using cesium chloride gradient centrifugation. Genome libraries were prepared using a GS FLX Titanium rapid library preparation kit and analyzed using a Roche/454 GS FLX Titanium system. Reads were quality checked and assembled using GS De Novo Assembler v2.8. The E. coli DH10B sequence was rejected *in silico* using GenomeMatcher v3.0 (9). The genome was annotated using Microbial Genome Annotation Pipeline (MiGAP) v2.23 (10). Default parameters were used except where otherwise noted.

Sequence data were obtained for a region of 74,277,843 to 131,327,361 bp from 131,990 to 232,733 reads (Table 1). The four plasmid sequences were assembled into 18 to 71 contigs, comprising 83,862 to 141,617 nucleotides at 516× to 878× coverage (G+C content, 50.69 to 53.06%). The four plasmid sequences were annotated to contain 93 to 208 putative coding sequences, including 5 to 20 genes related to antimicrobial resistance determined *in silico*.

**TABLE 1:** Summary of data for four large plasmids carrying antimicrobial resistance genes

Parameter	Data for plasmid:			
	p5	p15	p41-3	p41-5
Results of sequencing analysis				
Total no. of reads	153,363	232,733	131,990	141,597
Total read length (bp)	90,263,916	131,327,361	74,277,843	80,843,101
Total genome size (bp)	120,730	141,617	125,307	83,862
G+C content (%)	52.7	50.69	50.99	53.06
No. of coding sequences	135	208	150	93
Coverage (fold)	516	833	539	878
No. of contigs	34	71	22	18
Maximum contig length (bp)	31,488	30,084	30,525	37,533
GenBank accession no.	LC318090 to LC318123	LC317979 to LC318049	LC318050 to LC318071	LC318072 to LC318089
DRA accession no.	DRR213731	DRR213732	DRR213733	DRR213734
Putative Inc groups	FIA, FIB	F, N	F, N	FIA, FIB
Antimicrobial resistance genes				
No. of genes	16	20	10	5
Detected genes (no. of copies)	*bla*_TEM-1_, *strA*, *strB*, *aphA1* (3), *aacC3* (2), *tetA* (2), *cat-20*, *aac6′-lb-cr*, *sul1*, *sul2*, acetyltransferase, streptomycin 3′-*O*-adenylyltransferase	*bla*_CTX-14_ (3), *strA*, *strB*, *ant3′9*, *tetA* (5), *tetC*, *tetO* (4), *teTR_D*, *dhfr*, *sul1*, *sul2*	*bla*_TEM-1_, *bla*_CTX-14_, *ant3′9*, *tetA* (2), *tetC*, *tetO*, *teTR_D*, *dhfr*, *sul1*	*strA*, *strB*, *aacC3*, *tetA*, *sul2*

Antimicrobial resistance genes included on the four plasmids were well matched to the resistance gene profiles demonstrated using Southern hybridization in our previous study (8). The putative Inc groups for the four plasmids were any of the F, FIA, FIB, or N groups, all of which are known to have self-transmission abilities. In addition, mobile genetic elements such as transposons and insertion sequences were observed in the flanking regions of each antimicrobial resistance gene. These results indicate that a single event of horizontal transfer of a plasmid might generate a multidrug-resistant microorganism. Moreover, two plasmids, p41-3 and p41-5, which showed different antimicrobial resistance gene profiles, were identified from strain QD1-5-9, indicating that more than one multidrug resistance plasmid can exist within one cell. Considering that plasmids containing various resistance genes have an influence on the diversity and complexity of circulating antimicrobial resistance profiles, further plasmid analysis will provide a better understanding of the distribution, diversity, and mechanisms of the emerging multidrug-resistant strains.

### Data availability.

The GenBank accession numbers are given in Table 1. Raw sequence data were deposited in the DRA under BioProject accession number PRJDB9410.
